# Failing to learn the lessons: the U.S. response on global health security ignores 20 years of PEPFAR

**DOI:** 10.1002/jia2.26108

**Published:** 2023-06-01

**Authors:** Brian Honermann, Emily Bass, Greg Millett

**Affiliations:** ^1^ amfAR The Foundation for AIDS Research Andelson Office of Public Policy Washington DC USA; ^2^ Independent Scholar New York USA

1

Sometimes compared to the post‐war U.S. aid package for Europe, the Marshall Plan, the U.S. President's Emergency Plan for AIDS Relief (PEPFAR) is among the most successful U.S. foreign aid investments in the twenty‐first century. PEPFAR's well‐documented successes [1, 2, 3] include more than 20 million people currently receiving antiretroviral treatment, decreases in new HIV acquisition and AIDS deaths, as well as indirect impacts: increased economic growth, health workforce capacity, and enhanced laboratory and supply chain infrastructure, and several countries supported by PEPFAR already meeting UNAIDS 95‐95‐95 targets.

Despite bipartisan recognition of the programme's strengths and successes, the Biden Administration and Congress are considering changes to U.S. government global health programming that ignore PEPFAR's lessons and create risks for the programme. Specifically, the Department of State has proposed, and Congress is now considering, a reorganization of its global health functions into a new health bureau that paradoxically both elevates and—as proposed—undermines PEPFAR and its leadership.

The new health bureau would consolidate an array of global health work scattered across the State Department and put the U.S. Global AIDS Coordinator that directs PEPFAR, in charge, with an additional title: Ambassador‐at‐Large for Global Health Security and Diplomacy. Bringing coherence to State Department health efforts spanning HIV/AIDS, SARS CoV2, MPOX and more through a single bureau is a laudable goal. The PEPFAR programme and its head are excellent resources to build on. But the present proposal fails to ensure the core components of PEPFAR's success are protected for the AIDS response and extended to other endeavours. These components, explored below, are: consolidated leadership, clear accountability and adequate resources. There is time to revise the proposed approach, building on and preserving these core strengths.

**Consolidated leadership**: The U.S. Global AIDS Coordinator is a presidentially appointed, Ambassador‐level position housed within the office of, and reporting directly to, the U.S. Secretary of State. This provides the Coordinator with the highest levels of diplomatic authority. As importantly, the Coordinator has direct operating control over programming, including budgets, targets, metrics and coordination of global HIV activities across government agencies. While the State Department itself does not implement programming, it leverages the unique technical capacities of the appropriate agencies to implement.

By contrast, the proposed State Department reorganization assigns the Coordinator a new role that carries no budgetary, strategic or coordination authority, and more limited diplomatic authority. Moreover, recently passed legislation creates a muddled set of roles and processes for the USG global health security agenda. In addition to the new role at the State Department, the domestically focused PREVENT Pandemics Act (PREVENT Act) [4] and the internationally focused Global Health Security and International Pandemic Prevention, Preparedness, and Response Act of 2022 (GHS Act) [5] assign different responsibilities and authorities across the White House Office of Pandemic Preparedness, the Centers for Disease Control and Prevention (CDC), the United States Agency for International Development (USAID) and the State Department, without creating a coordinated structure to hold all programming together.

Global health security and pandemic prevention, preparedness and response (PPPR) are different from the ongoing response to established pandemics like HIV/AIDS, tuberculosis or malaria. There are effective treatments and prevention modalities for all three diseases; impact on new infections, illness and death can be directly measured and reported on. PPPR and health security encompass a wider array of disciplines, health workforce functions, diplomatic considerations and more.

However, PEPFAR's lesson about ensuring a fit‐for‐purpose structure for U.S. global health investments must be heeded, or else money and time may be wasted where none can be spared. Clarity about who is in charge and what is to be achieved with U.S. resources is both essential and missing.

**Accountability**: PEPFAR has set and met targets for two decades, including in the context of a decade of flat funding, because the programme is able to shift resources to programmes and approaches that work. People living with HIV, activists, health workers and government partners in PEPFAR‐funded countries shape, implement and deliver these services. But in Washington, DC, there is a single person—the U.S. Global AIDS Ambassador—who reports on the programme's performance to Congress, and who has the mandate to achieve results against annual plans. There is no comparable accountability for results in the proposed approach to global health security. Although the development of a strategy is in the works at the National Security Council, there are no signs that this strategy will be matched to a budget and coordinating authority that binds all agencies to a singular vision of what USG resources will achieve, and how impact will be measured. Absent this, particularly in a space where strategies and priorities can change dramatically between administrations and with broad mandates, such as “strengthening health systems,” the door is opened to fragmentation that achieves few practical results.

**Resources**: Finally, PEPFAR helped transform the course of a global pandemic because it delivered funding—albeit belatedly—that was commensurate to the need. The GHS Ambassador and office in the State Department will not have a budget for implementation, nor will any other single entity.

The President's Budget request of $1.245 billion for Global Health Security brings the lack of cohesion into sharp focus [6]. The request seeks an appropriation that would be largely carved up among USAID, bilateral and multilateral funds. USAID would be funded $450 million for all bilateral programming, while the State Department's allocation of $500 million is to be passed on to The Pandemic Fund at the World Bank, essentially providing the new Ambassador and Health Bureau nothing but an unfunded mandate and extremely limited authority. Fragmented resources and diffuse leadership do not drive strong implementation.

Congress and the Administration should re‐think the approach building on the lessons from PEPFAR: establish empowered leadership to hold all programming to a consolidated vision for U.S. investments, create real accountability and buy‐in for programming in coordination with Congress, and appropriate meaningful levels of resources in a manner that enables implementation of the vision and accountability for its execution. In other words: learn from PEPFAR lest we lose time in preparing for the next pandemic.

## COMPETING INTERESTS

The authors have no competing interests.

## AUTHORS’ CONTRIBUTIONS

BH and EB contributed to primary drafting. BH, EB and GM all provided content and editing.

## FUNDING

No financial support for this piece.
